# Diphtheria in the WHO European Region, 2010 to 2019

**DOI:** 10.2807/1560-7917.ES.2022.27.8.2100058

**Published:** 2022-02-24

**Authors:** Mark Muscat, Belete Gebrie, Androulla Efstratiou, Siddhartha S Datta, Danni Daniels

**Affiliations:** 1 World Health Organization Regional Office Europe, Copenhagen, Denmark; 2 WHO Collaborating Centre for Reference and Research on Diphtheria and Streptococcal Infections, UK Health Security Agency, London, United Kingdom

**Keywords:** diphtheria, *Corynebacterium diphtheriae*, public health surveillance, epidemiology

## Abstract

**Background:**

Diphtheria is uncommon in the World Health Organization (WHO) European Region. Nevertheless, sporadic cases, sometimes fatal, continue to be reported.

**Aim:**

To report on diphtheria cases and coverage with first and third doses of diphtheria, tetanus and pertussis vaccines (DTP1 and DTP3, respectively) for 2010–19 in the Region with a focus on 2019.

**Methods:**

Data on diphtheria cases were obtained from WHO/United Nations International Children's Emergency Fund (UNICEF) Joint Reporting Forms submitted annually by the Region’s Member States. WHO/UNICEF Estimates of National Immunization Coverage for DTP1 and DTP3 were summarised for 2010–19. For 2019, we analysed data on age, and vaccination status and present data by country on DTP1 and DTP3 coverage and the percentage of districts with ≥ 90% and < 80% DTP3 coverage.

**Results:**

For 2010–19, 451 diphtheria cases were reported in the Region. DTP1 and DTP3 coverage was 92–96% and 95–97%, respectively. For 2019, 52 cases were reported by 11 of 48 countries that submitted reports (including zero reporting). Thirty-nine countries submitted data on percentage of their districts with ≥ 90% and < 80% DTP3 coverage; 26 had ≥ 90% districts with ≥ 90% coverage while 11 had 1–40% districts with < 80% coverage.

**Conclusion:**

Long-standing high DTP3 coverage at Regional level probably explains the relatively few diphtheria cases reported in the Region. Suboptimal surveillance systems and inadequate laboratory diagnostic capacity may also be contributing factors. Still, the observed cases are of concern. Attaining high DTP3 coverage in all districts and implementing recommended booster doses are necessary to control diphtheria and prevent outbreaks.

## Introduction

Diphtheria is an acute bacterial disease caused by *Corynebacterium* species. The most common type of diphtheria is classic respiratory diphtheria caused by toxin-producing *Corynebacterium diphtheriae*. The disease is characterised by a membranous inflammation of the upper respiratory tract, with widespread damage to other organs, primarily the myocardium and peripheral nerves. *C. diphtheriae* is transmitted by physical contact via respiratory secretions from a patient or a carrier. Most diphtheria-related deaths result from the effects of the toxin and include acute systemic toxicity, myocarditis and neurologic complications. The case fatality of respiratory diphtheria is 5–10% even with treatment [1]. Non-toxigenic strains may cause a sore throat but do not produce membranous lesions. Less commonly, diphtheria affects the skin (cutaneous diphtheria) and mucous membranes at other non-respiratory sites, such as genitalia and conjunctivae [2]. Two other potentially toxigenic species, *Corynebacterium ulcerans* and *Corynebacterium pseudotuberculosis*, are primarily zoonotic infections but can also cause disease in humans. *C. ulcerans* infection is associated with disease indistinguishable from that caused by toxigenic strains of *C. diphtheriae* [3,4].

Following the massive re-emergence of diphtheria in the newly independent states of the former Soviet Union (NIS) in the 1990s [5], the disease is currently considered uncommon in the World Health Organization (WHO) European Region. Nevertheless, sporadic cases, sometimes resulting in death, continue to be reported.

We hereby present data on diphtheria for 2010–19 with a focus on 2019. We also report on coverage with a diphtheria-containing vaccine represented by first and third doses of diphtheria, tetanus and pertussis vaccines (DTP1 and DTP3, respectively). Data on vaccination schedules, and school-based screening and vaccination activity for 2019 are also presented.

## Methods

Data on the number of reported diphtheria cases for 2010–19 (as at 6 April 2021) were obtained from WHO/United Nations International Children's Emergency Fund (UNICEF) Joint Reporting Forms (JRFs) submitted to the WHO Regional Office for Europe. This form has been in use since 1998 and is intended to collect countries’ annual immunisation data through a standard questionnaire sent to all 53 Member States of the WHO European Region [6]. Only the reported total cases of diphtheria were considered. These comprised laboratory-confirmed cases, epidemiologically linked cases and clinical cases; suspected cases of diphtheria were not included in the analysis. Since 2018 the JRF requests that all toxigenic diphtheria cases should be reported. It specified that asymptomatic, mild, cutaneous, and mucosal and respiratory cases should be included if laboratory confirmed as toxigenic diphtheria and that non-toxigenic diphtheria cases should be excluded. Also, since 2018, the JRFs allows for the collection of data on cases by age group and vaccination status. 

For 2019, we analysed the data by age and vaccination status obtained in the JRF for that year (as at 6 April 2021). We also report on data (as at 6 April 2021) on vaccination schedules, and school-based screening and delivery of routine doses of vaccines on the national immunisation schedule to children at school (excluding doses of vaccine given in supplementary immunisation activities or other vaccination campaigns) obtained in the same JRF. Where no data on school-based screening and vaccination activity for 2019 was provided, we used data from the JRF for 2018. School-based screening refers to the routine checks of a child’s vaccination status at the time of enrolment to or during primary and secondary school. We considered primary school to begin at 5–7 years of age, with a typical duration of 4 to 6 years, and secondary school to begin usually around 14–15 years of age, with a typical duration of 4 years [7].

Data on diphtheria-related deaths were obtained for 2010–19, except for 2016, for which no request had been made in the JRF for that year. Case fatality was calculated on diphtheria-related deaths as a percentage of the number of cases reported for the 9 years for which data on deaths had been requested in the JRFs.

WHO/UNICEF Estimates of National Immunization Coverage (WUENIC) for DTP1 and DTP3 coverage (as at 4 October 2021) [8] were summarised for 2010–19. In addition, DTP1 and DTP3 coverage, and the percentage of districts with ≥ 90% and < 80% DTP3 coverage for 2019 were presented by country. We considered the first three doses of DTP-containing vaccine as the primary series; subsequent doses were considered booster doses. Percentages were rounded to the nearest whole number.

### Ethical statement

We did not seek an ethical evaluation of this work as no personal data were collected. The data presented in this article are based on WHO/UNICEF JRFs submitted annually by Member States of the WHO European Region.

## Results

### Diphtheria cases

**Figure 1:**
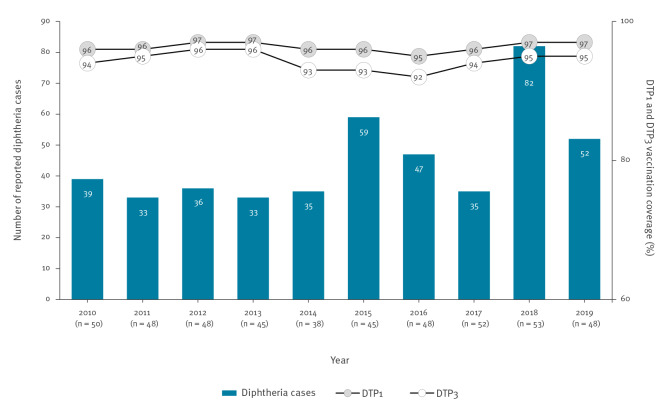
Number of reported diphtheria cases^a^ (n = 451) and DTP1 and DTP3 coverage in the WHO European Region, 2010–2019

DTP1: first dose of diphtheria, tetanus and pertussis vaccine; DTP3: third dose of diphtheria, tetanus and pertussis vaccine; WHO: World Health Organization.

^a^ The number of countries that submitted reports (including zero reporting) on diphtheria cases are shown in parentheses below the year.

For 2010–19, the number of countries that submitted reports (including zero reporting) ranged from 38 in 2014 to 53 in 2018. During this 10-year period, there were 451 cases of diphtheria reported in the Region (Figure 1). For 2019, the number of countries that submitted reports (including zero reporting) was 48 of which 11 countries, altogether, reported 52 diphtheria cases: Germany (n = 15), United Kingdom (UK) (n = 12), Belgium (n = 6), Russian Federation (n = 5), Sweden (n = 4), Latvia (n = 2), Norway (n = 2), Slovakia (n = 2), Spain (n = 2), Georgia (n = 1) and Greece (n = 1). For the 9 years for which data on deaths was available, there were 12 diphtheria-related deaths reported from six countries: Latvia (n = 5 deaths), France (n = 2), UK (n = 2), Greece (n = 1), Spain (n = 1) and Turkey (n = 1). This gives a case fatality in the Region of 3%.

**Figure 2:**
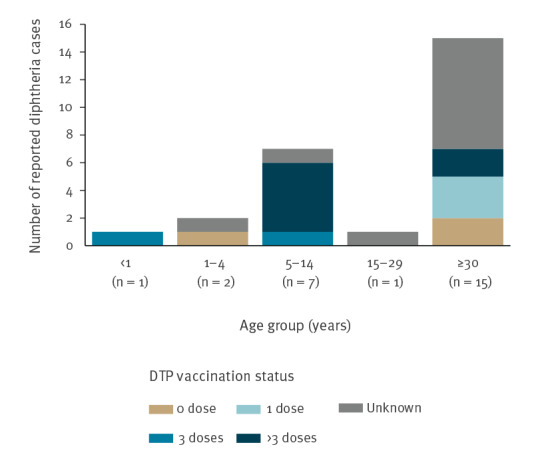
Diphtheria cases by age and DTP vaccination status in the WHO European Region, 2019 (n = 26)^a^

DTP: diphtheria, tetanus and pertussis; WHO: World Health Organization.

^a^ Excluded from the graph are the 26 cases with no data on age. These include 17 cases that also had no data on vaccination status.

Of the total 52 reported cases in 2019, 26 cases had data on age: one case was aged < 1 year, two were aged 1–4 years, seven were aged 5–14 years, one was aged 15–29 years and 15 were aged ≥ 30 years. The vaccination status was known for 24 cases. Of these, seven were unvaccinated, five received one dose, two received three doses and 10 received > three doses. For the remaining 28 cases, the vaccination status was unknown and included 17 cases that also had missing data on age. Figure 2 shows the age distribution of cases by vaccination status.

There were no reported diphtheria cases with two DTP doses.

Of the total cases, 46 cases (88%) were laboratory-confirmed. These were reported by nine countries and were mostly from Germany (n = 15) and the UK (n = 12) (Table).

**Table:** Number of diphtheria cases, DTP1 and DTP3 coverage, district level DTP3 coverage, timing of third dose, number of booster doses for diphtheria-containing vaccine, and school-based screening and vaccination activity by country, WHO European Region, 2019

Country (n = 53)	Diphtheria cases		Vaccine coverage (%)		Per cent of districts with DTP3 coverage:		Age at third vaccine dose (months)	Booster doses (n)	Routine screening of vaccination status at:		Delivery of routine doses of vaccines at school
	Total (n = 52)	Laboratory-confirmed (n = 46)	DTP1	DTP3							
					≥ 90%	< 80%			Primary school	Secondary school	
Albania	0	0	99	99	100	0	≤ 6	≥ 3	Yes	ND^a^	No
Andorra	0	0	99	99	NA	NA	> 6	2	Yes	Yes	No
Armenia	0	0	96	92	84	0	≤ 6	≥ 3	No	No	No
Austria	0	0	90	85	ND	ND	> 6	1	ND^a^	ND^a^	Yes
Azerbaijan	0	0	96	94	87	5	≤ 6	2	Yes	Yes^c^	No
Belarus	0	0	98	98	100	0	≤ 6	≥ 3	Yes	Yes	Yes
Belgium	6	6	99	98	ND	ND	≤ 6	≥ 3	Yes	Yes	Yes
Bosnia and Herzegovina	0	0	89	73	65	12	≤6	2–3^b^	Yes^c^	Yes^c^	Yes^c^
Bulgaria	0	0	96	93	86	4	≤ 6	≥ 3	Yes	Yes	No
Croatia	0	0	98	94	90	0	≤ 6	≥ 3	Yes	Yes	Yes
Cyprus	0	0	98	96	100	0	≤ 6	≥ 3	Yes	Yes	Yes
Czechia	0	0	99	97	99	0	> 6	2	No	No^c^	No
Denmark	0	0	97	97	100	0	> 6	1	Yes	No	No
Estonia	0	0	92	91	73	0	≤ 6	≥ 3	Yes	Yes	Yes
Finland	0	0	98	91	ND	ND	> 6	≥ 3	Yes	Yes	Yes
France	ND	ND	99	96	ND	ND	> 6	≥ 3	ND^a^	ND^a^	ND^a^
Georgia	1	0	99	94	82	0	≤ 6	≥ 3	No^c^	No^c^	No
Germany	15	15	98	93	56	3	≤ 6	≥ 3	Yes	No^c^	No
Greece	1	1	99	99	ND	ND	≤ 6	≥ 3	Yes	Yes	No
Hungary	0	0	99	99	100	0	≤ 6	≥ 3	Yes	Yes	Yes
Iceland	0	0	95	92	ND	ND	> 6	2	Yes	No	Yes
Ireland	0	0	98	94	97	0	≤ 6	2	No	No	Yes
Israel	0	0	99	98	100	0	≤ 6	≥ 3	Yes	No	Yes
Italy	0	0	98	96	95	0	> 6	≥ 3	Yes	No	No
Kazakhstan	0	0	99	97	100	0	≤ 6	≥ 3	Yes	Yes^c^	Yes
Kyrgyzstan	0	0	99	95	95	0	≤ 6	≥ 3	Yes	Yes	Yes
Latvia	2	2	99	99	100	0	≤ 6	≥ 3	Yes	No	No
Lithuania	0	0	96	92	84	2	≤ 6	≥ 3	Yes	No	No
Luxembourg	ND	ND	99	99	ND	ND	≤ 6	≥ 3	Yes^c^	Yes^c^	No^c^
Malta	0	0	98	98	NA	NA	≤ 6	2	Yes	Yes	Yes
Monaco	0	0	99	99	NA	NA	> 6	1	Yes	Yes	No
Montenegro	ND	ND	94	85	ND	ND	≤ 6	≥ 3	Yes^c^	No^c^	Yes^c^
Netherlands	0	0	98	94	90	4	< 6 to > 6^d^	2	ND^a^	ND^a^	ND^a^
North Macedonia	ND	ND	98	92	ND	ND	≤ 6	≥ 3	Yes^c^	Yes^c^	Yes^c^
Norway	2	2	99	97	95	0	> 6	2	No	No	Yes
Poland	ND	ND	99	95	ND	ND	≤ 6	≥ 3	No^c^	No^c^	No^c^
Portugal	0	0	99	99	100	0	≤ 6	≥ 3	Yes	Yes	No
Republic of Moldova	0	0	91	91	68	11	≤ 6	≥ 3	Yes	Yes	No
Romania	0	0	96	88	45	10	> 6	2	No	No	No
Russian Federation	5	0	97	97	100	0	≤ 6	≥ 3	Yes	Yes	Yes
San Marino	0	0	90	88	NA	NA	> 6	≥ 3	Yes	Yes	No
Serbia	0	0	99	97	100	0	≤ 6	≥ 3	Yes	No	No
Slovakia	2	2	99	97	100	0	> 6	≥ 3	Yes	No	No
Slovenia	0	0	98	95	100	0	≤ 6	2	Yes	Yes	No
Spain	2	2	98	96	95	0	> 6	≥ 3	No	No	No^e^
Sweden	4	4	98	98	93	0	> 6	2	Yes	Yes	Yes
Switzerland	0	0	98	96	41	0	≤ 6	≥ 3	Yes	Yes	Yes
Tajikistan	0	0	98	97	100	0	≤ 6	≥ 3	Yes	Yes	No
Turkey	0	0	99	99	91	1	≤ 6	≥ 3	Yes^c^	No^c^	Yes
Turkmenistan	0	0	99	99	100	0	≤ 6	≥ 3	Yes	Yes	No
Ukraine	0	0	89	80	16	40	≤ 6	≥ 3	Yes	Yes	No
United Kingdom	12	12	97	93	81	1	≤ 6	2	Yes	Yes	Yes
Uzbekistan	0	0	96	96	100	0	≤ 6	≥ 3	Yes	Yes	Yes

DTP1: first dose of diphtheria, tetanus and pertussis vaccine; DTP3: third dose of diphtheria, tetanus and pertussis vaccine; NA: country that is not divided into subnational levels; ND: no data; WHO: World Health Organization.

^a^ No data was provided for 2019 and 2018.

^b^ Number of booster doses varies between the country’s three entities.

^c^ Based on 2018 data as no data for 2019 was provided.

^d^ In the Netherlands, the third primary dose is given at 4–11 months of age.

^e^ However, some regions of Spain administer the booster dose at 14 years of age in a school setting.

### DTP1 and DTP3 coverage

All 53 countries reported on DTP1 and DTP3 coverage for 2010–19. Coverage at Regional level was relatively stable during this period and, in 2019, was at 97% and 95%, respectively (Figure 1). For 2019, 32 countries reported ≥ 95% DTP3 coverage. The remaining 21 countries reported < 95% DTP3 coverage and included six countries reporting < 90% DTP3 coverage: Austria (85%), Bosnia and Herzegovina (73%), Montenegro (85%), Romania (88%), San Marino (88%) and Ukraine (80%).

For 2019, 39 countries submitted data on the percentage of districts with ≥ 90% and < 80% DTP3 coverage (Table). Of the remaining 14 countries, four countries did not report on these variables because they are not divided into subnational levels. Twenty-six countries achieved ≥ 90% DTP3 coverage in ≥ 90% of their districts. Twenty-eight countries reported none of their districts with < 80% DTP3 coverage. Of the remaining 11 countries: seven countries had 1–5% of their districts with < 80% DTP3 coverage, three countries had 10–12%, and one country had 40%.

### Vaccination schedules

In 2019, all 53 countries offered a primary series of three doses of diphtheria-containing vaccine. Fifteen countries recommended the third dose after 6 months of age (Table). One country recommended the third dose at 4–11 months of age.

All 53 countries gave at least one booster dose, with 37 countries providing the recommended three or more booster doses, 12 countries provided two booster doses and three countries provided one booster dose. One other country provided two to three booster doses depending on the subnational level.

### School-based screening and vaccination activity 

In 2019, most of the 53 countries (n = 42) reported that they routinely check the vaccination status of children at primary school. Of these, 30 countries also routinely check the vaccination status at secondary school (Table). Twenty-four countries reported the delivery of routine doses of vaccines on the national immunisation schedule to children at school.

## Discussion

Prior to the widespread use of diphtheria immunisation, the disease was a major cause of death among children [2]. Diphtheria is now considered uncommon in the WHO European Region; of the over 87,500 diphtheria cases reported globally in 2010–19 [9], only 451 cases were from the Region. For 2019, there was a decline in the total reported cases from 82 cases in 2018 to 52 cases. We assume that the 46 laboratory-confirmed cases of the 52 total cases in 2019 were all toxigenic diphtheria since, from 2018, the JRF specifically requested all such diphtheria cases to be reported, including cases presenting with respiratory, cutaneous and mucosal forms of the disease. Despite this decline, the reported cases are still of concern, highlighting the need for more efforts to address diphtheria in the Region.

The WHO Regional Office for Europe had set two targets related to diphtheria in its European Vaccine Action Plan (EVAP) [10]. The first (EVAP goal 4) was to achieve ≥ 95% DTP3 coverage at national level in 48 of the 53 countries (90%). The second (EVAP objective 4) was on geographical equity within the countries in which ≥ 90% districts (or equivalent administrative units) achieve ≥ 90% DTP3 coverage. Despite the reported high coverage at Regional level, these coverages were not consistent across the Region as for 2019, 21 countries had < 95% DTP3 coverage and included six countries reporting < 90% coverage. In addition, of the 39 countries in the Region that reported data by district, 11 countries had districts with < 80% DTP3 coverage, indicating geographical inequities in vaccination uptake that need to be addressed. One factor in preventing a major outbreak in a community is the herd immunity threshold which, for diphtheria, has been estimated at 80–85%, based on average age of infection in the pre-vaccine era [2]. To minimise the potential for diphtheria to re-emerge, population immunity through vaccination should be maintained at high levels in all areas. Screening of vaccination status at school entry can provide an effective opportunity to catch up on any missed vaccinations. Immunisation programmes targeting school-age children are increasingly important and particularly relevant for booster doses of diphtheria toxoid-containing vaccine.

Most diphtheria cases were reported in adults aged 30 years and older. This finding concurs with that reported in recent reviews of diphtheria epidemiology that showed an age distribution shift, with cases mostly occurring in adolescents and adults [11,12]. In countries where diphtheria has been well controlled and the disease has become sporadic, immunity is known to wane in late childhood or adolescence depending on the schedule of immunisation [2]. With diphtheria becoming uncommon, it can be assumed that there is little chance of exposure to infection that would provide natural boosting of immunity in adults following that induced by childhood immunisation. Most countries (n = 37) in the Region provide the recommended three or more booster doses of diphtheria toxoid during childhood and adolescence to compensate for the loss of natural boosting after completion of the primary immunisation series during infancy. However, nearly a third of the countries (n = 17) in the Region provided less than the three recommended booster doses. People living in low incidence or non-endemic areas may require booster doses of diphtheria toxoid at about 10-year intervals to sustain immunity following a three-dose primary and three-dose booster schedule before adolescence [13]. However, more recent data suggest that the administration of decennial booster doses following this schedule may not be necessary through middle age [14,15]. Nevertheless, this needs to be monitored in the long term given the increasing life expectancy worldwide [16].

Long-standing high coverage with DTP3 at Regional level is probably the main reason why there are relatively few diphtheria cases reported in the Region. Still, the cases observed in the Region are of concern, and may be also partly attributed to suboptimal surveillance systems and inadequate or lack of specialised laboratory diagnostic capacity. Indeed, sustaining the required laboratory capacity in countries particularly with zero or low incidence of diphtheria is a major challenge and significant gaps in this field of work has been reported in the Region [17,18]. The areas with significant gaps are related to training and surveillance of all three potentially toxigenic corynebacteria – *C. diphtheriae*, *C. ulcerans* and *C. pseudotuberculosis.* Surveillance systems should be in place for the three pathogens, with appropriate methods to determine toxigenicity. Early and accurate laboratory diagnosis of each suspected case is essential to inform proper treatment of a case and management of close contacts. In recent years, the WHO Collaborating Centre for Reference and Research on Diphtheria and Streptococcal Infections in collaboration with the WHO Regional Office for Europe [19] and the European Centre for Disease Prevention and Control (ECDC) have organised training workshops to improve diphtheria diagnostic capacity in 26 countries in the Region including 11 NIS.

### Limitations

Comparisons between countries should be made with caution because apart from potential differences in the quality of diphtheria surveillance, importation potential of cases may also vary among countries. Moreover, the JRF does not stipulate a common case definition and classification for countries to use. However, there are WHO-recommended surveillance standards for vaccine-preventable diseases in place to serve as a guide to good practice and may help to harmonise surveillance activities [20]. Furthermore, the data collected in the JRFs does not distinguish between respiratory diphtheria and non-respiratory presentations of the disease. We can only assume that the cases reported in the JRFs before 2018 were cases of respiratory diphtheria, since the WHO-recommended surveillance standards for diphtheria [21] – before their revision in 2018 – focused specifically on this disease presentation. However, we cannot exclude the possibility that, although less common, non-respiratory presentations such as cutaneous disease may have been included among the reported cases especially since, in the last decade, cutaneous forms of the disease have been reported more frequently [4]. Indeed, collecting data on all clinical presentations of diphtheria is important to monitor changes in the epidemiology of the disease. The revised WHO-recommended surveillance standards invite countries to expand the case definition of suspected diphtheria cases to include non-healing ulcers in a person with a travel history to countries with endemic disease or countries with diphtheria outbreaks. It also recommends the collection of clinical data elements including cutaneous lesions and other non-respiratory involvement.

The lack of data on *Corynebacterium* species type limits the description of diphtheria epidemiology as does the lack of information on whether cases have been imported from abroad or acquired indigenously. The revised WHO surveillance standards recommend the collection of data on *Corynebacterium* species type, i.e. *C. diphtheria, C. ulcerans* and *C. pseudotuberculosis*, as well as travel history within 10 days of onset of illness.

The relatively small number of cases reported annually cautions against interpreting significant epidemiological trends. Data on age and vaccination status was only available for 15 cases out of the total 52 reported cases in 2019. Another limitation is that data on diphtheria-related deaths was restricted to the number of reported fatal cases without information on key variables such as age and vaccination status. In addition, a request for data on diphtheria-related deaths for 2016 had not been made in the JRF for that year so the case fatality rate of 3% could only be calculated using 9 of the 10 years of the study period. An additional case of diphtheria reported to WHO Regional Office from Belgium in 2016 and later published in the literature [22] was therefore excluded from the calculation.

### Recommendations

Although reported DTP1 and DTP3 coverage rates at Regional level were maintained at a high level throughout 2010–19, for 2019, 11 countries had districts with < 80% DTP3 coverage. Countries should strive to ensure strong national immunisation programmes that address geographical inequities in vaccination uptake.

While all countries offered a primary series of three doses of diphtheria-containing vaccine, 17 countries provided less than the three recommended booster doses. Immunisation programmes should ensure that three primary doses and three booster doses of diphtheria toxoid-containing vaccine of age-appropriate formulations with respect to potency are provided during childhood and adolescence. Possible options for giving booster doses are: at the age of 12 months, at the age of primary school entry and a third booster dose on completion of primary school or start of secondary school [15]. A WHO-convened expert group on the use of reduced diphtheria toxoid (≥ 2–5 IU) has concluded that tetanus-diphtheria with reduced diphtheria toxoid (Td) vaccines currently licensed for ages 7 years and older, can be given in ages 4–7 years, as a second booster dose. This use of Td would be beneficial for immunisation programmes in many low- and middle-income countries [23].

To monitor the epidemiology of toxigenic diphtheria more closely, data collection on different clinical presentations and *Corynebacterium* species causing the disease is recommended. For the WHO European Region, a request for data collection on these variables will be included in future JRFs.

Although reports of diphtheria are uncommon in the Region, both clinicians and laboratory personnel should maintain a high index of suspicion in patients presenting with signs and symptoms of respiratory or cutaneous diphtheria, particularly after being in countries endemic for the disease. Indeed, surveillance systems for this disease including laboratory diagnostic capacity need to be adequate to ensure that cases are not missed. All countries are urged to undertake national surveillance primarily to monitor disease burden and identify outbreaks. The WHO surveillance standards for vaccine-preventable diseases provides guidelines that countries should consider in establishing and improving existing surveillance of such diseases, including that of diphtheria [20]. All providers identifying cases should be required to report them and, if possible, laboratory testing of all suspected cases should be conducted for case confirmation. An adequate surveillance of diphtheria requires that laboratories are equipped with the appropriate materials and that all isolates of potentially toxigenic corynebacteria should ideally be submitted to a reference/specialist laboratory for confirmation of identification and toxigenicity testing. A revised WHO manual for laboratory diagnosis of diphtheria and related infections has recently been published to assist laboratory workers in the correct procedures to diagnose diphtheria cases and to guide clinicians in treatment options [24].

## Conclusion

The relatively few diphtheria cases reported in the Region are probably the result of overall long-standing high DTP3 coverage at Regional level. However, attaining high DTP3 coverage in all districts and implementing recommended booster doses are necessary to maintain control of diphtheria and prevent outbreaks. At the same time, surveillance systems for this disease also need to be optimal and laboratory diagnostic capacity adequate to ensure that cases of toxigenic diphtheria are not missed.
